# Parental beliefs and practices around child weight in London, England: insights from qualitative photo-elicitation interviews

**DOI:** 10.1093/pubmed/fdag031

**Published:** 2026-04-24

**Authors:** Emily H Emmott, Grace Bronstone, Viktoria Möslinger-Gehmayr, Leah de Souza-Thomas, Jackie Chin

**Affiliations:** UCL Anthropology, University College London, 14 Taviton Street, London WC1H 0BX, UK; UCL Anthropology, University College London, 14 Taviton Street, London WC1H 0BX, UK; UCL Anthropology, University College London, 14 Taviton Street, London WC1H 0BX, UK; Office for Health Improvement and Disparities, Department for Health and Social Care, 39 Victoria Street, London SW1H 0EU, UK; Office for Health Improvement and Disparities, Department for Health and Social Care, 39 Victoria Street, London SW1H 0EU, UK

**Keywords:** childhood obesity, childhood overweight, health communication, parenting, qualitative research

## Abstract

**Background:**

Childhood overweight and obesity remain global public health concerns, yet caregiver engagement with prevention and support services is often limited. Understanding caregiver beliefs and everyday practices may help improve communication and service uptake.

**Methods:**

Seventeen parents from diverse backgrounds across London, England, participated in autophotographic photo-elicitation interviews exploring daily routines. Interview data were analysed using interpretive critical discourse analysis to identify norms and beliefs surrounding children’s weight and weight-related behaviours.

**Results:**

Five discourses were identified: (i) ‘Food at the Centre of Weight’; (ii) ‘Body Mass Index (BMI) as an Indicator, not a Diagnosis’; (iii) ‘Homecooked Food as Nourishment’; (iv) ‘Negotiating Challenges for a ‘Balanced Diet’; and (v) ‘Activity for Enjoyment and Development—not Health’. Parents viewed children’s weight as primarily shaped by food and diet, and rejected BMI as a standalone measure of healthy weight. Dietary practices centred on balancing nourishing home cooking with less nourishing foods, while physical activity was valued for its developmental benefits.

**Conclusions:**

Caregiver discourse revealed tensions between everyday norms and dominant public health messaging around children’s weight. In England, communications may be strengthened by acknowledging the limitations of BMI categories, resisting diet-centric framings, and aligning more closely with caregiver perspectives on food and activity.

## Introduction

Globally, childhood overweight/obesity is associated with poorer childhood wellbeing [1–3] and later-life morbidity/mortality [4–6]. In England, in 2022, 27% of children aged 2–15 years were classified as overweight or obese [7]. Childhood overweight/obesity rates are notably high in some areas of London, and in 2023/24, seven of the 32 London Boroughs reported overweight/obesity prevalence above 40% among Year 6 children (typically aged 10–11 years) [8].

The WHO promotes regular child growth monitoring for early detection of potential health issues including overweight/obesity [9]. In England, primary-school children are measured at Reception (4–5 yrs) and Year 6 (10–11 yrs) as part of the National Child Measurement Programme (NCMP), and parents/caregivers are informed of their children’s Body Mass Index (BMI) category. Set-up in 2005/2006 to monitor trends in childhood overweight/obesity, the NCMP is now widely used by local authorities (local government responsible for local public health) to identify children with potential overweight/obesity. Families are then referred to weight management services (typically a 12-week behavioural weight management programme) [10, 11]. However, uptake is generally low. An evaluation of eight family weight management programmes in England found that only 51% of referrals led to enrolment. Of those who enrolled, only 37% completed the programme [10]. Research suggests that families from minority ethnic backgrounds, lower socio-economic positions, and single-parent families are less likely to participate in weight management programmes [12].

Despite continued efforts, childhood overweight/obesity rates among primary-school children in England have remained static over the last 20 years [13]. Understanding how caregivers approach children’s weight may reveal pathways to improve engagement with weight management support. Studies show that mothers of children who are overweight or obese typically do not perceive them as being too heavy [7], and parents may react to BMI information with lack of trust, dismissal, and anxiety around child wellbeing [14, 15], suggesting careful framing of children’s weight is required. In the West, parenting and overweight/obesity are highly moralized and scrutinized [16–18], meaning effective communication must negotiate sensitive cultural norms and parental beliefs.

## Aims

Through photo-elicitation interviews, this study explored caregiver norms and beliefs around children’s weight and weigh-related behaviours in London, and considered their implications for practice. This study was conducted in partnership with the Office for Health Improvement and Disparities London.

## Methods

### Ethics and consent

This study was approved by the UCL SHS Anthropology Ethics Committee (ref: SHSAnth-2324-055-1). All participants provided informed consent to take part on a voluntary basis.

### Data collection

We conducted autophotographic photo-elicitation interviews to explore and understand broader family routines, with particular focus on weight-related behaviours. Participants were eligible if they were aged 18 or older, co-resided (including part-time) with child/ren aged 4–12 years within Greater London, and had caregiving responsibilities for the child(ren). Recruitment was conducted opportunistically through multiple channels to promote participant diversity and ensure representation from both Inner and Outer London (see Table 1 and SI).

**Table 1:** Participant characteristics.

ID	Recruitment pathway	London Bourgh (Outer/Inner)	Participant background information
EE1	Survey	Waltham Forest (Outer)	The participant is a White woman, presumed to be British, in her 40s. She lives with her Polish husband and 12-year old daughter. They bought their house and moved into a residential area of Waltham Forest ‘before it got gentrified’ 12 years ago. The participant recently returned to full-time employment in marketing. Her partner also works full-time, mostly from home. [Higher managerial, administrative and professional occupation]
EE2	Survey	Hounslow (Outer)	The participant is a White woman aged 35–44 years. She lives with her two children, aged 3 yrs (in nursery, part-time) and 5 yrs (in school), and her partner who is from Barbados. The participant works part-time in health research administration. Her partner is primarily a stay-at-home parent, with additional income through rental properties. [Intermediate occupation]
EE3	Survey	Havering (Outer)	The participant is a White woman, aged 30, and works in university administration. She lives with her two daughters, aged 2 and aged 6, and her partner. Her partner has 2 older children from his earlier marriage, and they come to stay on some weekends. They live in a suburban area in a shared ownership home. She describes her neighbourhood -where she has lived most of her life- as a hard-working, working-class area. Her mother and younger sister both live nearby, and her mother provides regular after-school childcare. [Intermediate occupation]
EE4	Survey	Sutton (Outer)	The participant is a Mixed-raced woman aged 35–44 yrs, assumed to be British. She lives with her husband, their 9-year-old son, and her 16-year old son (who her husband is a step-father to). She works full-time from home for local government, and her partner works full time as a postman. They live in a leafy suburban area in an ex-council flat within a small council estate. [Intermediate occupation]
EE5	Survey	Islington (Inner)	The participant is a Black woman aged between 35–44 yrs, originally from Sierra Leone, who moved to Islington as an infant. She is currently training to be a midwife. She lives in her 1-bed council flat with her son, who is 4.5 yrs old (in nursery). Her partner/father of her son lives outside of London, but stays over frequently and provides childcare. A family friend also provides ‘out of hours’ childcare while the participant is undertaking work placements. Her immediate family has moved out of London. [Not in employment—student]
EE6	Social Media	Waltham Forest (Outer)	The participant is a White man, presumed to be British, in his 30s. He lives with his wife and two children (one in nursery, and one in primary school). He is a stay-at-home parent, and his wife works full time as an actuary. They moved into a residential area in Waltham Forest to ‘settle down’ a few years ago. He was born in London, and grew up in the Borough of Camden. His parents still live there, and provides semi-regular/ad-hoc childcare. [Not in employment—stay at home parent]
GB7	Survey	Newham (Inner)	The participant is a Black woman aged approximately 35–45 years. She lived most of her life in London and resided in Islington for 10 years before moving to Newham a few years ago. The participant currently lives with her husband, who works in accounting, and their 7-year-old son in primary school. The participant worked as a teaching assistant until her son was born, then she transitioned to a stay-at-home parent. [Not in employment—stay at home parent]
GB8	Weight Management Programme	Newham (Inner)	The participant is from Sri Lanka and moved to the UK approximately 13 years ago after her husband received a work visa. He currently works in Central London at Tesco. The participant lives in a flat with her husband and two daughters, aged 8 and 3.5 years. The participant studied English literature and is currently a stay-at-home parent, but plans to look for work as a teaching assistant after her youngest daughter starts nursery school. Her family remain back home in Sri Lanka. [Not in employment—stay at home parent]
VM9	Social Media	Kensington & Chelsea (Inner)	The participant is a White woman in her 50s and is the sole caregiver for her 9 yr old daughter. She has been living in the same flat on housing benefits for 22 years, and is well connected in the local community. She has a 23 yr old daughter who lives elsewhere. She is neurodivergent, with past addiction issues. Her child is also neurodivergent. She is an active member of Narcotics Anonymous and regularly provides support to new members. [Not in employment – stay at home parent]

(*Continued*)

**Table 1:** Continued

ID	Recruitment pathway	London Bourgh (Outer/Inner)	Participant background information
VM10	Social Media	Westminster (Inner)	The participant, originally from Mexico, is a racially mixed (mestizo) woman in her 40s. She initially moved to the UK for university, then met her husband who is from the UK. They have two children, aged 10 months and 6 years. She works in the civil service and is currently on maternity leave. They live in a flat which she describes as overcrowded, with the children sharing a bedroom and her husband working from home. Both her and her husband’s families live abroad or far away. [Intermediate occupation]
GB11	Weight Management Programme	Newham (Inner)	The participant, assumed to be in her 40s–50s and originally from India, moved to Newham over 15 years go for better work opportunities. She lives with her husband and 10-year-old daughter. Both the participant and husband works at a restaurant (cashier & waiter), and they alternate shifts to care for their daughter. The participant also makes and sells clothes as an additional source of income. Note, GB12 was also present during interview. [Routine & manual occupations]
GB12	Weight Management Programme	Newham (Inner)	The participant, assumed to be in her 50s and originally from India, moved to London approximately 15 years ago. She lives in a home in Newham with her three children and husband. Her oldest son (aged 19) attends a local college, her daughter (aged 14) attends secondary school, and her son (aged 9) attends primary school. She works as a manager of a restaurant. Note, GB11 was also present and assisted with translation. [Higher managerial, administrative and professional occupation]
VM13	Leaflet	Westminster (Inner)	The participant is a White woman, aged 32, who has lived in the borough of Westminster most of her life, and currently resides in a council flat. She has two children, a 13-year-old-son and a 5-year-old-daughter. Her partner, who works late shifts, lives separately but stays over frequently. The participant works part time at a soft play/playgroup. Her partner’s family provides significant support, especially his sister and father. The participant has a wide social network of friends from different areas. [Routine & manual occupations]
EE14	Snowball via EE5	Hackney (Inner)	The participant is a Black woman in her 30s and a single parent to her daughter aged 4 in preschool. She grew up in Suffolk and moved to Hackney approx. 25 yrs ago, and has lived in the same flat for 20 yrs near the local high street. She works part-time as a social worker, and part-time on her own counselling business. She does not have family nearby, as they still live in Suffolk. She has minimal contact with her ex-partner, and his family live overseas in Tobago. She has a strong network of friends who she spends time with regularly on weekends. [Higher managerial, administrative and professional occupation]
VM15	Leaflet	Westminster (Inner)	The participant is a Black woman aged 28 yrs who lives in a council flat in Westminster. She was born locally and grew up in the area. The participant is a single parent and has two children; a four-year-old son and a one-year-old daughter. She is currently a stay-at-home parent due to unaffordable childcare costs, but is planning to return to work as a housing officer. She receives support from her mother, the children’s father, and friends. [Not in employment—stay at home parent]
VM16	Leaflet	Westminster (Inner)	The participant is a White woman in her 40s from the United States, who moved to the UK in 2012 for university. She is married to a Lebanese man who is a wheelchair user. They have two daughters, aged nine and five. She lives in social housing (two-bedroom with small garden) in Westminster, and works part-time and remotely for an international charity based in London. She used to work full-time, but reduced her hours after she was diagnosed with cancer (now in remission). Her and her husband’s family live overseas. [Intermediate occupation]
VM17	Leaflet	Islington (Inner)	The participant is a Black man aged 47 yrs who moved to London from Eritrea when he was 13. His wife is also from Eritrea, and they have four daughters aged 11, 8, 4 and 0. They live in a small two-bedroom council flat, but they are hoping to move to a three or four bedroom flat soon. He works in the area of project management/business consulting, and is also a company director. His wife is currently a stay-at-home parent, but plans to return to work in the future. His immediate family members live overseas. [Higher managerial, administrative and professional occupation]

The participant ID indicates who conducted the interview. Characteristics of each London borough can be viewed at https://trustforlondon.org.uk/data/boroughs/. Participant occupation types are noted within the participant background information, categorised according to the National Statistics Socio-Economic Classification.

In total, 18 individuals across nine London boroughs were recruited and interviewed between March and July 2024. During one interview, it became clear that the participant did not have caregiving responsibilities, rendering them ineligible. The final sample therefore comprised 17 eligible participants.

Three interviewers (EHE, GJB, and VMG) conducted data collection using a shared interview protocol/guide. Interviews were held in participants’ homes or public spaces (e.g. cafés, libraries) and were audio-recorded. Prior to the interview, participants were asked to ‘capture their days’ by photographing a typical weekday and weekend (2 days total) on their smartphone.

During interview, these photographs were used to reconstruct and map out a timeline of their daily routines. Interviewers asked unstructured follow-up questions to elicit motivations and contextual detail, with a focus on weight-related family behaviours (e.g. children’s activities, food consumption). Interviews typically lasted 1–1.5 hours. Participants formally transferred between 0 and 17 photographs to researchers (mean = 6.4), with some informally showing additional images. As a token of thanks, participants received a £20 shopping voucher after the interview.

Note, this single data collection supported three related but distinct projects. As a result, there were some variations in the focus of unstructured follow-up questions between interviewers (see SI). Nonetheless, all participants (i) mapped out family routines, (ii) discussed weight-related behaviours, and (iii) were asked about their views on measuring children’s BMI.

### Sample characteristics

Participant backgrounds which were self-disclosed during data collection are outlined in Table 1. Of the 17 participants, 15 were mothers and 2 were fathers. Five participants (29%) resided in Outer London (comparatively suburban), and 12 participants (71%) resided in Inner London (comparatively urban). Where participants disclosed their age, most were in their 30s and 40s. Reflecting the diversity of London, the self-disclosed heritage/ethnicities of participants varied, and 10 participants (59%) were identified as being from minorities ethnic backgrounds. The socioeconomic and household situations also varied in terms of employment, home ownership, and household composition—with four participants (23.5%) identified as being in managerial/professional occupations, five participants (29.4%) in intermediate occupations, two participants (11.8%) in routine/manual occupations, and five participants (35.3%) not currently employed. The majority of participants did not self-declare their educational qualifications.

### Analysis

Data analysis was conducted by EHE, who took a structuralist perspective recognizing that individual behaviours and experiences must be understood in the context of wider societal systems. Following Ecological Systems Theory, child development (including overweight/obesity) was viewed as being influenced by multiple factors across multiple levels of life/society. Guided by these theoretical assumptions, EHE conducted an interpretive critical discourse analysis of the interviews [19](see SI). Here we assume that participant language and narratives reflect societal power relations, structural constraints, cultural norms and personal beliefs. The analysis sought to uncover the deeper meanings embedded in participants’ accounts, moving beyond literal interpretations, and consider its implications. Through the analysis process, emerging findings were sense-checked and discussed with all project members. Further information on the analysis, including coding and validation methods, are available in the SI.

## Results

We identified five relevant discourses mapped onto two overarching topics (Table 2). Relating to ‘The Meaning of Children’s Weight’, we identified two discourses: ‘Food at the Centre of Weight and BMI as an Indicator, not a Diagnosis’. Relating to ‘The Drivers of Weight Related Behaviours’, we identified three discourses: ‘Homecooked Food as Nourishment, Negotiating Challenges for a ‘Balanced Diet,’ and ‘Activity for Enjoyment and Development—not Health’. We elaborate on each discourse below.

**Table 2:** Summary of findings outlining main topics, discourses, finer discursive elements and number of relevant cases.

Topic	Discourse	Number of cases with discourse present	Number of cases where relevant topic was not discussed (i.e. information missing)	Number of negative cases (i.e. data conflicts with discourse)
The Meaning of Children’s Weight	Food at the centre of weight	12	5	0
	BMI as an indicator, not a diagnosis	12	3	2^a^
The Drivers of Weigh-Related Behaviours	Homecooked food as nourishment	15	1	1^b^
	Negotiating challenges for a ‘balanced diet’	16	1	0
	Activity for enjoyment and development – not health	15	2	0

Further details on negative cases are as follows: (a) GB11 and GB12 (note, they were interviewed in the presence of each other), where participants were accepting of BMI as accurate, although they were broadly not interested in the measure. It is important to note that they were recruited via a weight management programme where weight/BMI is measured weekly; (b) GB11, where participant indicated they had not really thought about nutrition until they recently started cooking lessons via a weight management programme. During interview, food was not discussed in terms of nourishment, but in terms of enjoyment. Food options were primarily driven by children’s preferences and costs.

### Discourse 1: Food at the Centre of Weight

While participants explicitly acknowledged that children’s weight is shaped by multiple factors, conversations about weight consistently centred on food, with some explicitly expressing their belief that diet is the primary determinant of children’s weight:

**‘Interviewer:** […] do you think [children’s weight] is down to the food they eat, or like activities they are doing?’

**‘EE2:** It’s probably a bit of both, but it’s definitely going to be more of a diet. […] I mean, from my own experience, because I’ve always had weight issues, like… it’s definitely down, down to diet. I think fitness is good to have. […] But food, what you eat, counts.’

Entrenched social norms linking food and weight shaped participant narratives, often overriding contradictory personal experiences. GB12, for example, described her 9-year-old son’s recent weight loss through football. Yet, when her daughter raised feeling body-conscious, the participant’s advice focused on dietary control:

**‘GB12:** I used to ask her, what is your food habit? Because I know she's a foodie. So I asked her food habit. Do more activities… food habit… Drink lot of water. Water will digest. Water is the only remedy, no?’

Dieting was therefore positioned as a necessary pathway for children to transition out of overweight/obesity. When discussing the NCMP, some participants expressed concern about triggering unhealthy attitudes toward food, body consciousness, and poor mental health—emphasizing that children should be shielded from idealized body norms. This stood in tension with public health advice promoting ‘healthy weight,’ which implicitly reinforces ‘the ideal body.’ Such discomfort was particularly strong among a subsample of UK-born mothers who grew up through the ‘heroin chic’ diet culture of the 90s/00s, when extreme thinness and restrictive eating were glamourized [20].

This food-centric framing of children’s weight conflicts with current evidence that, unlike adults, diet-based interventions are not effective at preventing overweight/obesity for children (although there may be other health benefits) [12, 21]. However, it remains deeply embedded in both family and institutional contexts, including in public health. For example, EE4 shared her experience of attending a weight management programme for children with her eldest son. She described how the programme focused on ‘healthy eating,’ which she viewed as ineffective and harmful. They stopped attending after the first few sessions:

**‘EE4:** It was just basically the food advice they give on the NHS website. Which I don’t think it’s great anyway, because it seems very carb-heavy and… the NHS promotes Slimming World and stuff like that which I don’t think is healthy. I think that promotes a bad relationship with food. I don’t agree with that at all. […] it wasn't telling me anything I didn't know already. I think it probably just gave him more anxiety about [his weight], not that he ever said.’

Overall, this discourse suggests that public health initiatives addressing children’s weight should resist food-centric messaging. This requires challenging the dominance of diet culture by reframing food as just one of many factors influencing children’s weight, placing greater emphasis on supporting physical activity- in line with current evidence [12, 21].

### Discourse 2: BMI as an indicator, not a diagnosis

Participants recognized health as multifaceted and complex, and viewed BMI categories as insufficient for capturing children’s physical development. Thus, BMI was viewed as an ‘indicator’ rather than a ‘diagnosis’.

Parental perceptions of BMI’s usefulness varied—particularly in relation to how it was applied within the NCMP. When asked about the NCMP, some participants embraced the uncertainty of BMI categories, seeing this ambiguity as central to its usefulness. Rejecting BMI as a diagnosis, BMI was a piece of information to be considered alongside other factors when monitoring children’s health.

**‘VM17:** You know, they can give different body sizes… [BMI] does not give you an absolute, but gives you some sort of indications […] So it would be good, just to see. Yeah, and to follow up on that.’

Where participants viewed their children’s body composition to be genetically/developmentally determined, children’s BMI categories were dismissed as irrelevant—at least for their own children. Several participants from minority ethnic backgrounds specifically questioned the validity of BMI thresholds, stating that overweight/obesity cut-offs are standardized against White bodies.

**‘GB7:** I can't overfeed him. He can only eat as much as his stomach can take. And in terms of height, I can't do anything. He’s got the genes he’s got. And for this age, it doesn't really matter.’

It was notable that mistrust of BMI categories was often linked to participants’ past experiences with healthcare. Several described instances of medical measurements being applied with unwarranted certainty, leading to poor outcomes, eroding trust. For example, when discussing the NCMP, EE4 stated that she had ‘nothing against it personally,’ but she was ‘not bothered’ by it—reflecting her dismissal. She went on to explain how her ambivalence stemmed from her pregnancy experiences: in both pregnancies, her babies were measured as very large, and inductions were recommended. However, after a difficult birth experience with her first child—who turned out ‘not’ to be large—she refused induction for her second birth. This led to a better birth experience, and her second child was also average in size. This experience left her sceptical about medical growth measurements, and exemplifies how practices around medical measurements in pregnancy/birth can have long-lasting impact.

Overall, participants did not reject BMI because of its uncertainty. Instead, discomfort relating to children’s BMI categories stemmed from how imprecise measures were applied with undue certainty, effectively ‘diagnosing’ children as overweight or obese based on a single, context-free measurement. Initiatives like the NCMP may therefore benefit from explicitly framing BMI as a contextual indicator with some uncertainty, and engaging parents in dialogue that acknowledges the measure’s limitations.

### Discourse 3: Homecooked food as nourishment

Participants consistently described home-cooked meals as ‘healthy,’ with many making deliberate efforts to expose their children to a diverse range of foods. Diversity in diet, particularly through fruits and vegetables, was widely prioritized and viewed as essential to children’s growth and development. In this framing, ‘nourishment’ for the developing body was the central criterion for healthy eating.

**‘Interviewer:** …what’s, like, the main drivers of [choosing] certain things to cook?’

**‘EE14:** A balanced diet. Really, so, just making sure we are getting as much, sort of, just the right vitamins as best as I can. […] I just want to make sure she’s eating a varied diet as best as possible. So that’s why it’s really important for me to cook.”

In contrast, processed and pre-prepared foods (including takeaways) were described as ‘unhealthy,’ often due to concerns around synthetic preservatives, excess sugar, and other undesirable ingredients. Because home-cooked meals were assumed to be nourishing by default, participants rarely discussed portion sizes or calorie content, and did not perceive their cooking practices as contributing to ‘unhealthy weight.’ These findings suggest that public health messaging which assumes inadequacy in homecooked meals—such as promoting ‘healthier swaps’ or ‘healthy recipes’—may be misaligned with caregiver norms.

### Discourse 4: Negotiating challenges for a ‘balanced diet’

Food decisions were often negotiated within families, primarily shaped by children’s preferences and constrained by practicalities such as time and ease. Several participants described making different meals for children to accommodate allergies or preferences, and managed the provision of these meals within busy schedules.

Rather than restricting ‘unhealthy’ foods entirely, participants focused on balancing nourishing and less nourishing foods. Particularly among participants with ‘picky eaters,’ some placed little restriction on children’s choices, allowing them to determine what they ate—while ‘balancing’ this through various tactics to encourage nourishment (such as offering fruit and vegetables) and limiting access to less nourishing foods (such as hiding snacks).

**‘VM13:** I do buy crisps and biscuits and stuff like that, which is not great. But anyway, and he'll eat a whole like, you know, multi pack of crisps. So, I have to like hide things from him. But I do also buy a lot of like fruit, so grapes... I make sure that in my dinner that I do always have some kind of vegetables.’

Overall, a ‘balanced diet’ was understood as a balance between nourishing and less nourishing foods, not a balance of healthy foods. Often, a balanced diet was achieved through negotiation within child-centric eating environments. This suggests that initiatives that focus on educating caregivers about healthy eating may not lead to change unless caregivers are supported with strategies to help children enjoy and prefer nourishing foods.

### Discourse 5: Activity for enjoyment and development—not health

While participants consciously acknowledged that exercise and physical activity are important components of health, their discourse around children’s activities was rarely framed in relation to health. Instead, caregivers discussed physical activity in terms of broader child development, essential for building skills, confidence, socialization, and personal growth.

Among those who spoke about their children’s participation in organized physical activities (such as swimming or dance classes), motivations were primarily centred on children’s enjoyment and skill development. Participants often went out of their way to ensure children took part in activities they enjoyed while also developing their abilities. Caregiver motivations for children’s activity therefore aligned more closely with educational and developmental goals rather than health-related ones.

**‘Interviewer:** …what’s the reason behind choosing gymnastics?’

**‘EE6:** […] it also comes down to like, the sort of the set-up… Like, there is a closer gymnastics class, but … we heard it’s more competitive. […] we just want [daughter] feeling more confident in herself, and it’s not necessarily about competition. It’s like, getting her to do forward rolls, and balance, and things like that. So, just sort of building up confidence skills.’

When encouraging physical activity in children, the effectiveness of messaging may improve by framing activity as beneficial for children’s development and enjoyment, rather than narrowly focusing on health.

## Discussion

### Main findings of this study

This study identified five discourses reflecting caregiver norms around children’s weight and related behaviours. ‘Food at the Centre of Weight’ captured how diet was positioned as the primary driver of children’s weight. In ‘BMI as an Indicator, Not a Diagnosis’, BMI was viewed as a limited tool, useful only when considered alongside other contextual factors. ‘Homecooked Food as Nourishment’ reflected the belief that home-cooked meals are inherently healthy. ‘Negotiating Challenge’s for a ‘Balanced Diet’ described how families balanced nourishing and less nourishing foods within child-led routines. Finally, ‘Activity’ for ‘Enjoyment and Development—Not Health’ showed that physical activity was valued for enjoyment and skill-building, with little emphasis on health outcomes.

### What is already known on this topic

In England, the BMI of primary-school children is monitored via the NCMP and sent to parents/caregivers. Many LAs use this information to identify overweight/obese children and provide targeted weight management support [11]. However, studies show parents reject or disregard NCMP/BMI information [15], and there is poor engagement with children’s weight management services [10]. Thus, caregiver norms/beliefs may conflict with current weight management initiatives.

### What this study adds

This study reveals potential conflict between caregiver norms/beliefs and how children’s BMI may be used in public health communications. The implications of our findings, specifically regarding engaging with families with children, are as follows:

1. When measuring and discussing children’s BMI, acknowledge the imprecise nature of the categories to align with caregiver expectations. Avoid using BMI as a simple diagnosis of overweight/obesity, but as a tool to consider and discuss health.
2. Challenge diet culture by reframing food as one of many factors influencing children’s weight. Alongside current evidence that diet-based interventions are not effective in preventing obesity for primary-school children [12, 20], increase emphasis on physical activity.
3. Frame food in terms of nourishment. Offer guidance on a nutritious diet rather than ‘healthy foods.’
4. Provide strategies to help children like/prefer nutritious options, rather than educating caregivers on healthy eating.
5. Frame physical activity as beneficial for children’s broader development and enjoyment, rather than health.

An example application of these findings to the 2024 NCMP caregiver letter can be found at https://osf.io/v27rk.

### Limitations of this study

Our findings may not be transferrable to rural or international settings, families with more marginalized experiences, non-parental caregivers, or those managing significant health challenges in children. As the study focused on everyday routines and normative beliefs, findings are most applicable to the prevention of childhood overweight/obesity, rather than the clinical management of obesity. Finally, the analysis was led by one researcher (EHE). While regular validation meetings/discussions were had, this increases the risk of bias.

## Data Availability

Published research data including interviewer notes, participant photographs and interview transcripts are available via https://osf.io/ewzxf/ under ‘Files.’
